# Relationship between Adsorption and Toxicity of Nephrotoxic Drugs in Microphysiological Systems (MPS)

**DOI:** 10.3390/mi14040761

**Published:** 2023-03-29

**Authors:** Ryohei Ueno, Masahiro Kuninori, Takumi Sumi, Ramin Banan Sadeghian, Yuji Takata, Azusa Iguchi, Masahiro Tsuda, Fumiyoshi Yamashita, Kentaro Ichikawa, Ryuji Yokokawa

**Affiliations:** 1 Department of Micro Engineering, Kyoto University, Kyoto 615-8540, Japan; 2 Toyo Seikan Group Holdings, Ltd., Yokohama 240-0062, Japan; 3 Graduate School of Pharmaceutical Sciences, Kyoto University, Kyoto 606-8501, Japan

**Keywords:** microphysiological system (MPS), cyclo-olefin polymer (COP), nephrotoxicity, drug adsorption, drug screening

## Abstract

Microphysiological systems (MPS) are an emerging technology for next-generation drug screening in non-clinical tests. Microphysiological systems are microfluidic devices that reconstitute the physiological functions of a human organ using a three-dimensional in vivo-mimicking microenvironment. In the future, MPSs are expected to reduce the number of animal experiments, improve prediction methods for drug efficacy in clinical settings, and reduce the costs of drug discovery. However, drug adsorption onto the polymers used in an MPS is a critical issue for assessment because it changes the concentration of the drug. Polydimethylsiloxane (PDMS), a basic material used for the fabrication of MPS, strongly adsorbs hydrophobic drugs. As a substitute for PDMS, cyclo-olefin polymer (COP) has emerged as an attractive material for low-adsorption MPS. However, it has difficulty bonding with different materials and, therefore, is not commonly used. In this study, we assessed the drug adsorption properties of each material constituting an MPS and subsequent changes in drug toxicity for the development of a low-adsorption MPSs using COP. The hydrophobic drug cyclosporine A showed an affinity for PDMS and induced lower cytotoxicity in PDMS-MPS but not in COP-MPS, whereas adhesive tapes used for bonding adsorbed a significant quantity of drugs, lowering their availability, and was cytotoxic. Therefore, easily-adsorbed hydrophobic drugs and bonding materials having lower cytotoxicity should be used with a low-adsorption polymer such as COP.

## 1. Introduction

Recently, physiological functions and drug toxicity have been evaluated using cells cultured in a microfluidic device [1]. Microfluidic devices reconstruct niches, which are microenvironments surrounding cells in vivo, by adding shear stress and different types of cells, greatly enhancing the physiological functions of the cultured cells compared to a conventional plate culture. Systems that reconstruct niches for higher physiological functions in cells are called microphysiological systems (MPSs). They are expected to improve the efficacy and relevance of clinical and non-clinical tests for drug screening [2,3,4]—especially the gut, liver, and kidney MPSs where drugs are absorbed, metabolized, and excreted. 

Different types of microfluidic structures exist in MPS. Bilayer microfluidic devices, which have two upper and two lower channels separated by a porous membrane, have been researched for over ten years [5]. These microfluidic structures correspond to a conventional two-compartmentalized well plate with cell culture inserts on a microscale and are suitable for modeling epithelial cell functions, such as absorption, metabolism, and excretion [6,7,8,9]. Moreover, the circulation of the culture medium provides cells with sufficient nutrients and shear stress to mimic the in vivo bloodstream and enhance physiological functions in MPS. Epithelial cells play an important role in the absorption and excretion of drugs. Their properties determine the bioavailability and clearance of drugs, which are significant parameters in drug screening tests. Therefore, remodeling epithelial cell functions in the MPS is a critical factor in achieving a precise prediction of drug efficacy in vivo. In addition, epithelial cells have cellular polarity, which is a structural difference between their apical and basolateral sides, and determines the transportation direction of drugs. In other words, the cytotoxicity and efficacy of drugs change depending on cellular polarity in vivo. Therefore, a bilayer MPS which enables peak epithelial cell functions and control of administration direction depending on cellular polarity is essential for high-throughput drug screening.

Polydimethylsiloxane (PDMS) is widely used in microfluidic devices because it enables the formation of microchannels through soft lithography and bonding with a porous membrane using the prepolymer PDMS as glue, a self-assembled monolayer such as 3-aminopropyltriethoxysilane, and oxygen plasma treatment [10,11,12,13]. However, PDMS is known to adsorb small hydrophobic molecules such as rhodamine B [14]. Moreover, some researchers investigated the absorption of different types of hydrophobic drugs into PDMS [15,16,17]. Since many drugs are small hydrophobic molecules, MPS made from PDMS has the problem of adsorption of drugs, even though surface treatments, such as oxygen plasma, prevent the adsorption onto PDMS [18]. Owing to this situation, PDMS-MPSs were evaluated for adsorption or absorption of metabolites and drugs [19,20]. Moreover, different polymers have been researched for the development of MPSs with low adsorption and absorption instead of PDMS [21]. In particular, cyclo-olefin polymers (COPs) have been considered for properties such as low hygroscopicity, low adsorption/absorption, and high transparency [22]. However, the bonding process in COPs is difficult. Generally, thermal and solvent bonding are used. However, to achieve sufficient bonding strength via thermal bonding, the bonding temperature should be higher than the glass-transition temperature of the COP. Therefore, thermal bonding causes deformation of the microstructures in the COP chip. Because solvent bonding must soften and deform the bonding interface of the COP chip, the deformation of the microchannels is a challenge [23]. Recently, surface-activated bonding technologies for COP have been developed, which enable low-temperature bonding of the microstructures without deformation [24,25]. However, they could not bond the COP microfluidic channels inserted with a porous membrane because the deformation of the bonding interface was too small to tightly close the gaps between the COP chips and membrane. Consequently, in the fabrication of the bilayer COP-MPS, an adhesive tape was used for the bonding.

In this study, we evaluated the adsorption and cytotoxicity of four nephrotoxic drugs using COP-MPS, in which the upper and lower channels were bonded using adhesive tape. First, the adsorption of the drugs was evaluated. Next, we compared the drug adsorption and cytotoxicity between COP-MPS and PDMS-MPS on renal proximal tubule epithelial cells (RPTECs) cultured in each MPS type.

## 2. Materials and Methods

### 2.1. Quantification of Drug Adsorption to Each Material Used in MPS

**Figure 1:**
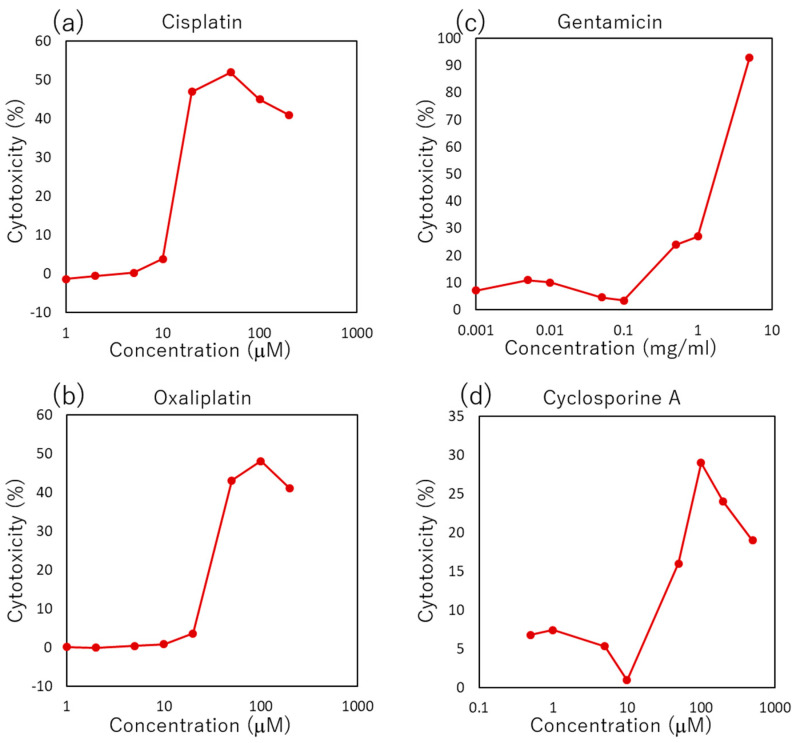
Concentration–cytotoxicity curve measured using the LDH assay kit after 96-h incubation. (**a**) Cisplatin; (**b**) Oxaliplatin; (**c**) Gentamicin; (**d**) Cyclosporine A.

The respective amounts of four nephrotoxic drugs adsorbed to each material used in an MPS were measured. The evaluated materials were COP, PDMS, a porous membrane (2000M12/580M303/XY, it4ip, Ottignies-Louvain-la-Neuve, Belgium), and an adhesive tape (9969, 3M, Minneapolis, MN, USA, described as Tape 0). Five slabs (5 mm diameter × 1 mm thickness) made of COP and PDMS were placed in microtubes (MS-4270M, Sumitomo Bakelite, Tokyo, Japan). The porous membrane was cut into 10 rectangles (5 mm × 14.3 mm), while the tape was cut into 11 (3 mm × 10 mm), before being placed in a tube. The size and the amount of each material were set to adjust the surface volume ratio of each material and medium to the ratio in a microchannel. Each material was incubated in 500 μL of renal epithelial growth medium (REGM, CC-3190, Lonza, Basel, Switzerland) containing a specific concentration of each nephrotoxic drug for 1, 2 and 4 h at room temperature with rotation at 1 rpm. At each time point, 100 μL of the solution was removed, and replaced with 100 μL of fresh medium containing the initial concentration of the drug. The initial concentration of each drug was set according to concentration–cytotoxicity curves as shown in Figure 1. To observe the reduction of cytotoxicity through adsorption, the concentration expressing a high level of cytotoxicity was avoided. Therefore, the concentration was adjusted to around the middle point between the beginning and the end point of the steep curve increasing cytotoxicity except cisplatin. For cisplatin, the concentration was adjusted to the same amount of oxaliplatin to compare the adsorption and the cytotoxicity. Considering these points, the concentrations were determined at 30 μM for cisplatin (033-20091, Wako, Osaka, Japan) and oxaliplatin (156-02691, Wako, Osaka, Japan), at 3 mg/mL for gentamicin (071-06453, Wako, Osaka, Japan), and at 50 μM for cyclosporine A (C2408, TCI, Tokyo, Japan), and kept consistent in this study. After the sampling, the concentrations of cisplatin and oxaliplatin were determined through ICP-OES. The concentrations of gentamicin were determined using LC-MS/MS. Quantification of cyclosporine A was performed by the Japan Testing Laboratory.

### 2.2. Cytotoxicity and Adsorptivity of Each Material Used in MPS

Changes in cytotoxicity caused by the adsorption of drugs to each material used in the MPS were measured using the lactate dehydrogenase (LDH) assay. To compare the adsorptivity and cytotoxicity of adhesive tapes, two different types of tapes (HJ-9150W, Nitto Denko, Tokyo, Japan, described as Tape A and NT-1001, As one, Japan, as Tape B) were tested. Each material was immersed in 500 μL of medium containing a specific concentration of each nephrotoxic drug, as described above, and incubated for four hours at room temperature with 1 rpm rotation. After incubation, each medium was collected, and applied to the RPTECs (CRL-4031, ATCC, Manassas, VA, USA) cultured in 96-well plates (167008, ThermoFisher, Waltham, MA, USA). After 96 h incubation without any replacement of medium, the cytotoxicity was determined using an LDH assay kit (CK12, Dojindo, Kumamoto, Japan). Moreover, to evaluate the cytotoxicity by the solvent in adhesive tapes, each tape was immersed into 500 μL of medium without any drug and incubated as described above. After incubation, each medium was collected and the cytotoxicity was measured. COP and PDMS slabs were not evaluated because they were not expected to have cytotoxic substances eluted to the medium.

### 2.3. Fabrication of MPS

The COP-MPS was fabricated via injection molding and bonded using adhesive tape. To compare adsorption, PDMS-MPS was fabricated using soft lithography and bonded using pre-polymer PDMS. Silicone tubes were bonded onto each inlet and outlet of the medium reservoirs using glue (RTV, KE-45, Shin-Etsu, Tokyo, Japan).

### 2.4. Cell Culture in MPS

Before cell seeding, each microfluidic channel was washed with 70% ethanol and filled with REGM after rinsing with sterilized water. To promote cell adhesion, an FNC coating mix (AES-0407-50, Athena Environmental Science, Baltimore, MD, USA) was introduced into the top channel and incubated for one minute. After removing the coating solution, a cell suspension (5 × 10^6^ cells/mL) was introduced and incubated overnight. After the cells had adhered onto the porous membrane, the medium was replaced with a fresh batch every two days.

### 2.5. Immunostaining

After a seven-day cell culture, the medium was removed from the devices, and the RPTECs were fixed in a 4% PFA solution for 15 min at room temperature. After rinsing with PBS, the cells were permeabilized with a solution of 0.05% Triton-X in PBS. After incubation with blocking buffer (PBS with 10% donkey serum) for one hour, cells were rinsed and incubated with primary antibody overnight at 4 °C. The cells were rinsed and incubated with a secondary antibody and DAPI in the dark for 2 h at room temperature. After rinsing, the samples were observed under a confocal laser scanning microscope (FV3000; Olympus, Tokyo, Japan). The antibodies used are listed in Table 1.

### 2.6. Comparison of COP-MPS and PDMS-MPS Cytotoxicity

Renal proximal tubule epithelial cells were seeded into COP- and PDMS-MPS as described above. On day 1, the confluent layer of RPTECs was confirmed and each channel was provided with 300 μL of fresh medium. On days 3, 5, 7, and 10, 200 μL of medium was removed from the top channel and the same amount of fresh medium was added. The sample solution was divided into two parts. One part was used for the quantification of kidney injury molecule-1 (KIM-1) using an ELISA kit (ADI-900-226-001, ENZO, New York, NY, USA) and the other was used for the quantification of LDH.

### 2.7. Comparison of Nephrotoxic Drug Cytotoxicity in COP-MPS and PDMS-MPS

Renal proximal tubule epithelial cells were seeded into COP- and PDMS-MPS and cultured for seven days. After confirming the confluent layer and refreshing the medium in the bottom channel, each nephrotoxic drug was introduced into the top channel and incubated for 96 h without any replacement of medium. The initial concentration was 30 μM for cisplatin and oxaliplatin, 3 mg/mL for gentamicin, and 50 μM for cyclosporine A. After 96 h, the cells were stained with Hoechst33342 (346-07951, Dojindo, Kumamoto, Japan), Annexin-V (A13201, Thermo Fisher, Waltham, MA, USA), and PI (P378, Dojindo, Kumamoto, Japan) to evaluate drug cytotoxicity by counting the number of apoptotic and dead cells.

## 3. Results

### 3.1. Quantification of Drug Adsorption to Each Material Used in MPS

**Figure 2:**
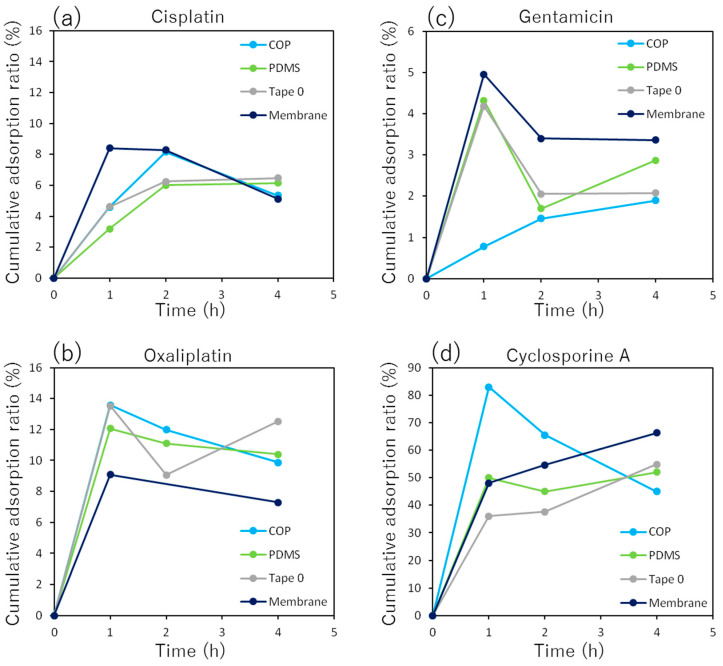
Time course of each nephrotoxic drug adsorption onto each material used in MPS. (**a**) Cisplatin; (**b**) Oxaliplatin; (**c**) Gentamicin; (**d**) Cyclosporine A.

The amount of the drugs adsorbed increased with incubation time and saturated each material within four hours (Figure 2). However, the pairing between some materials and drugs decreased over time. This phenomenon was possibly caused by the release of adsorped drug molecules from the materials. Generally, there are reversible and irreversible adsorptions. If the reversible adsorption was dominant, the decrease of adsorption is possible through the release of drug molecules depending on equilibrium state change. The amount of cisplatin adsorbed was 6–8% for all materials. At two hours, the COP and porous membranes had adsorbed more cisplatin than PDMS. However, at four hours, PDMS had adsorbed more.

The amount of oxaliplatin adsorbed was 8–14% in each material, which was approximately twice that of cisplatin. The adsorbed amounts were similar in both COP and PDMS. The time course of adsorption to the adhesive tape was unstable and reached a maximum at the end. On the other hand, adsorption to the porous membrane was the minimum in every material.

The amount of gentamicin adsorbed was 5% or less for each material and was the lowest for nephrotoxic drugs. Adsorption to COP increased with time and reached approximately 2% at the end. On the other hand, adsorption to all materials except COP rose sharply at the beginning and subsequently fell to approximately 2–3%.

The amount of adsorbed cyclosporine A reached up to 50–80% in every material and was the largest among all the drugs used in this study. Adsorption to COP increased sharply at the beginning and then gradually decreased to approximately 40%. On the other hand, adsorption to all the materials other than COP increased over time and reached 50–60% after four hours.

### 3.2. Cytotoxicity and Adsorptivity of Each Material Used in MPS

**Figure 3:**
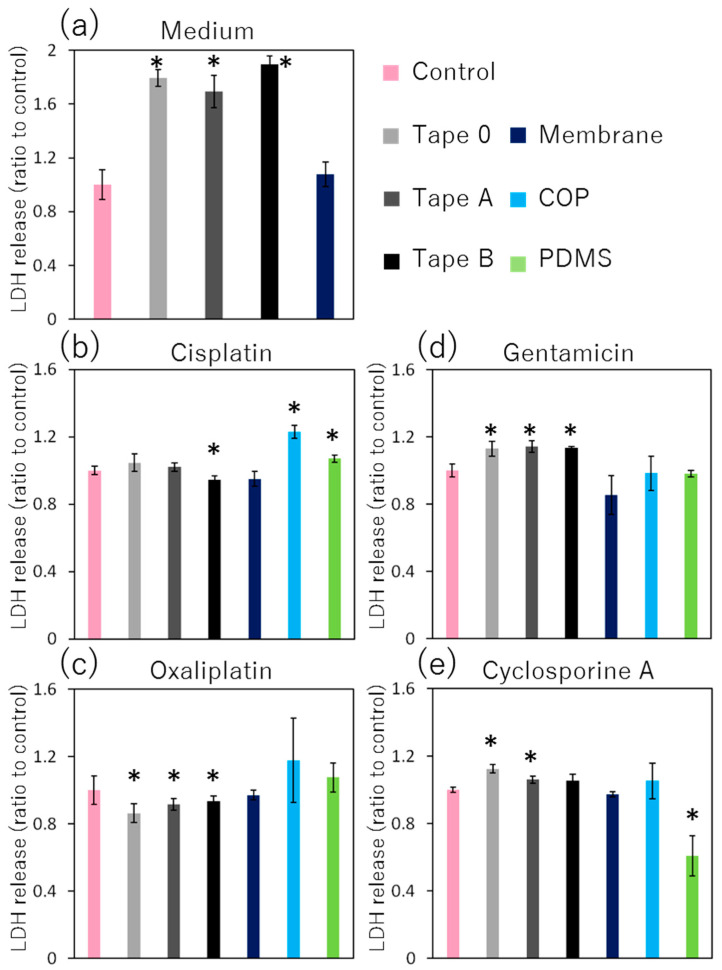
Cytotoxicity and drug adsorptivity of each material estimated by LDH release. Data are presented as mean ± SD (technical replicates, *n* = 3). * *p* < 0.05 against control. (**a**) Medium; (**b**) Cisplatin; (**c**) Oxaliplatin; (**d**) Gentamicin; (**e**) Cyclosporine A.

To examine the reduction of cytotoxicity through the adsorption of drugs onto each material, the amount of LDH was compared to a control sample, which was immersed with no material. Each medium immersing of three different types of adhesive tape showed cytotoxicity without any drugs. However, the medium that immersed the porous membrane did not show any cytotoxicity (Figure 3a). In the case of medium containing cisplatin, the cytotoxicity of Tape B decreased, but that of COP and PDMS increased compared to a control sample, which was immersed without any materials (Figure 3b). In the medium containing oxaliplatin, the cytotoxicity of every type of adhesive tape decreased, but that of the COP, PDMS, and porous membranes did not change (Figure 3c). In the case of medium containing gentamicin, every type of adhesive tape showed increased cytotoxicity; however, COP, PDMS and the porous membrane showed no such changes (Figure 3d). In the medium containing cyclosporine A, the cytotoxicity of Tape 0 and Tape A slightly increased, but that of PDMS decreased dramatically (Figure 3e).

### 3.3. Cell Culture in MPS and Immunostaining

**Figure 4:**
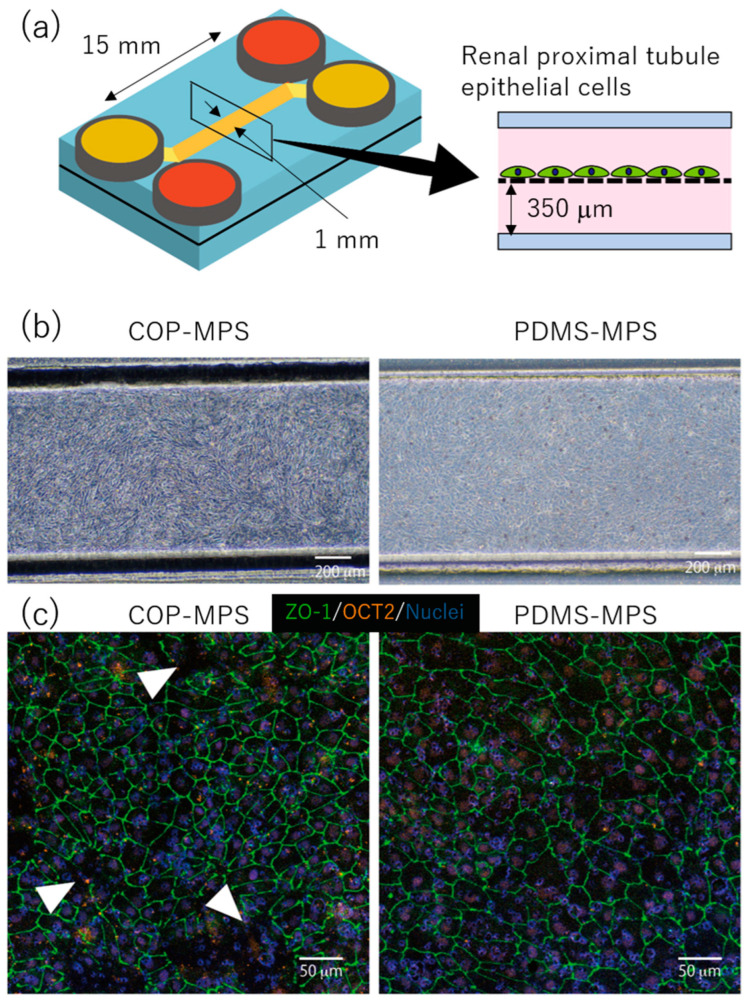
RPTEC cultured in COP-MPS and PDMS-MPS. (**a**) Schematic diagram of MPS structure; (**b**) Phase-contrast images of RPTEC; (**c**) Immunostaining images of ZO-1 (green), OCT-2 (orange), and nuclei (blue). White arrowheads indicate faint signals of ZO-1.

The RPTECs were seeded onto the upper channels of COP-MPS and PDMS-MPS as described above (Figure 4a). In both systems, RPTEC reached confluence on day 7 (Figure 4b). Immunostaining results of organic cation transporter 2 (OCT2) and zonula occludens-1 (ZO-1) in RPTEC cultured in each MPS were compared (Figure 4c). The expression of OCT2 was similar in both systems. On the other hand, ZO-1 expression was different between COP and PDMS (Figure 4c). In PDMS-MPS, ZO-1 was continuously expressed at the boundaries of each cell, and the integrity of the epithelial barrier was clear. However, in COP-MPS, ZO-1 was partially absent and the epithelial barrier was not perfect (Figure 4c, white arrowheads). 

### 3.4. Comparison of COP-MPS and PDMS-MPS Cytotoxicity

**Figure 5:**
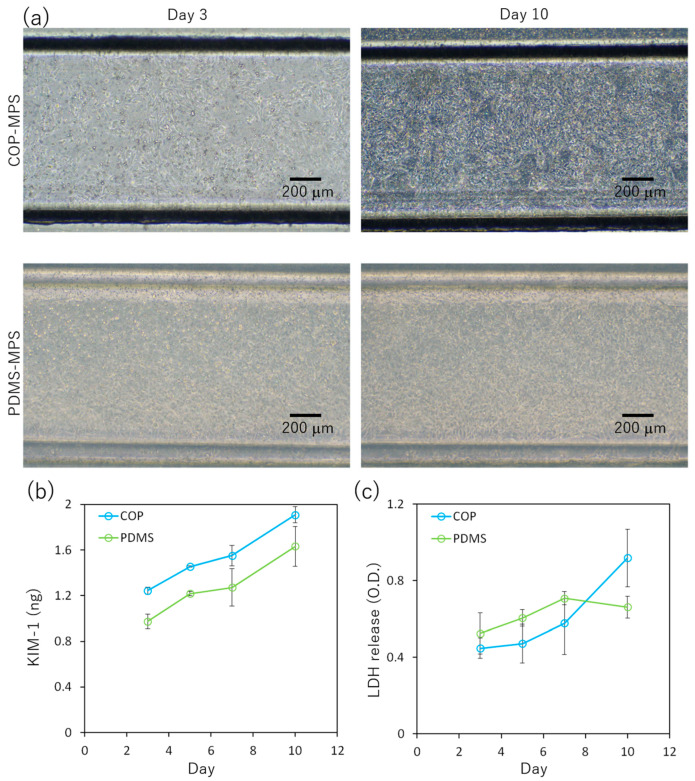
Comparison of cytotoxicity between COP-MPS and PDMS-MPS. (**a**) Phase-contrast images of RPTEC layers; (**b**) Time course of KIM-1 secretion; (**c**) Time course of LDH release. Data are presented as mean ± SD (technical replicates, *n* = 3). * *p* < 0.05.

Figure 5a shows RPTEC cultured in COP-MPS and PDMS-MPS on days 3 and 10. In both systems, RPTEC reached confluence on day 10; however, the amount of KIM-1 released from RPTEC in COP-MPS was slightly higher than that in PDMS-MPS, indicating that COP-MPS had greater cytotoxicity (Figure 5b). The amount of LDH from RPTEC in COP-MPS was lower than that in PDMS-MPS until day 7. However, on day 10, the amount in COP-MPS increased sharply (Figure 5c).

### 3.5. Comparison of Nephrotoxic Drug Cytotoxicity in COP-MPS and PDMS-MPS

**Figure 6:**
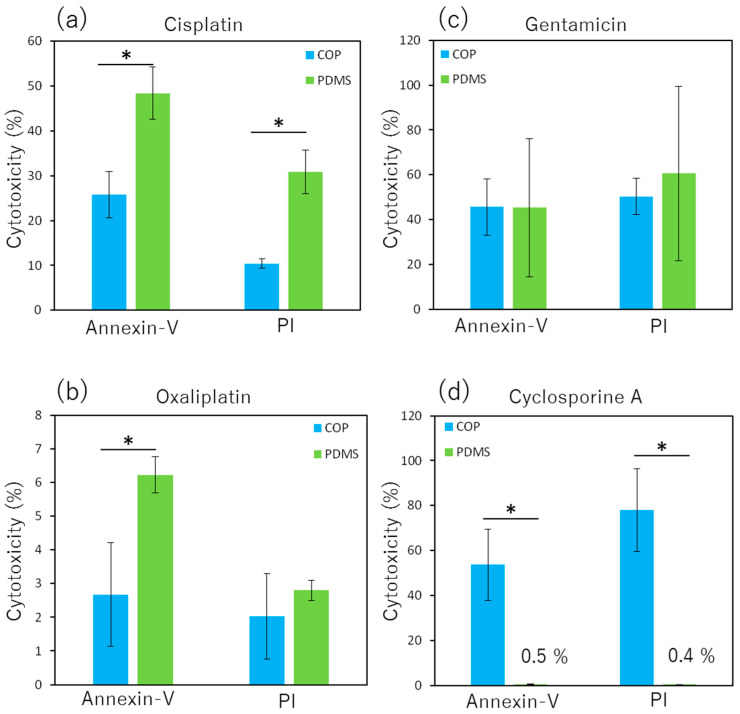
Cytotoxicity of each nephrotoxic drug on RPTEC cultured in COP-MPS and PDMS-MPS. (**a**) Cisplatin; (**b**) Oxaliplatin; (**c**) Gentamicin; (**d**) Cyclosporine A. Data are presented as mean ± SD (technical replicates, *n* = 3). * *p* < 0.05.

Cisplatin showed greater cytotoxicity toward RPTEC in PDMS-MPS than in COP-MPS (Figure 6a). However, oxaliplatin and gentamicin did not show any such differences in either system (Figure 6b,c). Cyclosporine A exhibited a dramatic reduction in cytotoxicity in PDMS-MPS compared with that in COP-MPS (Figure 6d).

## 4. Discussion

### 4.1. Quantification of Drug Adsorption to Each Material Used in MPS

In this study, cisplatin, oxaliplatin, gentamicin, and cyclosporine A were the nephrotoxic drugs selected. The molecular weight and partition coefficient (log P) of each drug are shown in Table 2. Partition coefficient indicates the ratio of solubility of drug in water and oil. Thus, cyclosporine A is the most hydrophobic among all four drugs because of the largest coefficient. Therefore, as shown in Figure 2d, the highest adsorption amount of Cyclosporine A was observed in every material used in MPS, since hydrophobic molecules had tendency to be easily adsorbed to a hydrophobic surface, such as polymers.

In a comparison between cisplatin and oxaliplatin, the amount of adsorption to the porous membrane was 5–8% for both (Figure 2a,b). On the other hand, the adsorption of oxaliplatin to the other materials was approximately twice that of cisplatin. Oxaliplatin is more hydrophobic than cisplatin owing to its larger partition coefficient. Thus, the higher amount of oxaliplatin adsorbed. Gentamicin has the smallest partition coefficient among the four drugs and is the most hydrophilic. Therefore, it had a lower tendency to be adsorbed to the polymers, and the results agreed with this (Figure 2c). 

### 4.2. Cytotoxicity and Adsorptivity of Each Material Used in MPS

Each culture medium, when immersing the three types of adhesive tape (Tape 0, Tape A, and Tape B), exhibited high cytotoxicity (Figure 3a). These results indicate that the substance eluted from the adhesive tapes was toxic. To reconstitute the cell culture environment in the MPS, the surface volume ratio of each material and medium was adjusted to the ratio in a microchannel. Consequently, the eluted substance is possibly cytotoxic in the microchannels as well.

In the case of cisplatin, Tape B showed decreased cytotoxicity (Figure 3b). As described above, adhesive tape is cytotoxic. However, the tape can adsorb cisplatin. Since the reducing effect of cisplatin exceeded the cytotoxicity of the eluted substance from the tape, the total cytotoxicity in Tape B decreased compared to that in the control sample. On the other hand, in the case of COP and PDMS slabs, the amount of adsorption to these materials was small and cytotoxicity slightly increased compared to that in the control. In the case of oxaliplatin, the cytotoxicity of every adhesive tape decreased compared with that of the control (Figure 3c). As described above, oxaliplatin is more hydrophobic than cisplatin, and adsorbs more to the adhesive tape. Thus, the adsorption of oxaliplatin to each tape decreased the concentration of oxaliplatin in the medium and its cytotoxicity. On the other hand, in case of gentamicin, cytotoxicity of every adhesive tape increased (Figure 3d). Gentamicin is a hydrophilic drug that has a low tendency for adsorption. The amount of adsorbed gentamicin was 5% or less, as shown in Figure 2c. Therefore, none of the materials showed a decrease in cytotoxicity caused by the adsorption of gentamicin. However, the adhesive tapes showed an increase in cytotoxicity caused by the substance eluted from the tapes. Being hydrophobic, cyclosporine A was expected to be adsorbed to every tape. However, two types of tape (Tape 0 and Tape A) increased the cytotoxicity, whereas PDMS decreased it drastically (Figure 3e). As a result, cyclosporine A was not adsorbed to adhesive tapes in the expected amount. However, PDMS adsorbed cyclosporine A and significantly decreased cytotoxicity.

### 4.3. Cell Culture in MPS and Immunostaining

The RPTECs were cultured in COP-MPS and PDMS-MPS. In both systems, the RPTECs formed a confluent layer and expressed ZO-1 (Figure 4b,c). In vivo, the epithelial layer separates the inside and outside of the body and forms a physical barrier between the epithelial cells for proper transportation. This barrier is called a tight junction, and ZO-1 is a protein that constitutes the tight junction. In this study, the structure of ZO-1 differed between COP-MPS and PDMS-MPS (Figure 4c). In PDMS-MPS, ZO-1 appeared uniformly on cell–cell boundaries in a confluent monolayer, and the barrier structure of the tight junctions was complete. On the other hand, in COP-MPS, ZO-1 partially disappeared and the barrier structure was not complete. These differences were attributed to the substance eluted from the adhesive tape used in COP-MPS for the bonding process. In other words, the toxicity of the adhesive tape inhibited the formation of tight junctions in RPTEC.

The OCT2 is a transporter protein that takes up cationic drugs such as cisplatin and oxaliplatin to excrete xenobiotics from the blood into the urine. Since RPTEC cultured in both MPSs expressed OCT2, the cytotoxicity caused by the uptake of cationic drugs through OCT2 was successfully evaluated. The basic technology for predicting nephrotoxicity using MPSs was demonstrated in this study.

### 4.4. Comparison of COP- and PDMS-MPS Cytotoxicity

In the COP-MPS group, tight junctions in RPTECs were only partially formed and did not have a sufficient barrier structure (Figure 4c). The cytotoxicity of the adhesive tape used in the COP-MPS for bonding may have inhibited the formation of the tight junctions. Therefore, to compare the basic cytotoxicity of COP-MPS and PDMS-MPS, conditioned medium was collected from each MPS, and cytotoxicity markers such as KIM-1 and LDH were measured in each collected sample. According to the results, COP-MPS expressed slightly higher cytotoxicity than PDMS-MPS (Figure 5). Given the toxicity of the eluted substance from the adhesive tapes (Figure 3a), it was probably the cause of the drastically impaired function of cells in COP-MPS. Moreover, lower gas permeability of COP should have negative effects on cell culture. On the other hand, the adsorption of proteins including growth factors in medium onto PDMS should have negative effects on cell. Thus, there are several factors to be evaluated in each MPS to reach a clear conclusion. Therefore, PDMS-MPS bonded through the same tape should have been tested to clearly verify the toxicity of the eluted substances.

### 4.5. Comparison of Nephrotoxic Drug Cytotoxicity in COP-MPS and PDMS-MPS

Cytotoxicity of each nephrotoxic drug was evaluated using COP-MPS and PDMS-MPS to compare the cytotoxicity change caused by drug adsorption to each system (Figure 6). Cisplatin cytotoxicity was greater in PDMS-MPS than in COP-MPS. Because cisplatin is hydrophilic, the reduction in cytotoxicity caused by adsorption is relatively small. However, the adsorption of cisplatin to the adhesive tape in COP-MPS possibly caused a reduction in cytotoxicity compared to that in PDMS-MPS (Figure 6a). On the other hand, cytotoxicity of oxaliplatin was too small to show any difference between COP-MPS and PDMS-MPS (Figure 6b). Gentamicin caused sufficient cytotoxicity. However, as described above, it was not absorbed by any of the materials. Therefore, no differences were observed between the systems (Figure 6c). Since cyclosporine A was easily adsorbed to PDMS because of its hydrophobicity, the cytotoxicity dramatically decreased in PDMS-MPS compared to that in COP-MPS. These results are in agreement with those shown in Figure 3e. As shown in Figure 6, the error bars are large and there are two possible explanations. One is that the initial concentration of drugs was moderate and the relatively low concentration should be reflected in the results. The other is in regard to the dilution of drugs in the MPS during incubation. The drugs were applied into the top channel but not the bottom channel. Therefore, after disruption of monolayer, the medium should be mixed between two channels through the porous membrane and the drug concentration should decrease. However, the disruption and mixing should not be uniform. Thus, one-side application of drugs should cause a deviation in the results. Cytotoxicity assay protocols using MPS should be assessed and discussed more since MPS has complex structures and functions compared with a conventional culture dish.

## 5. Conclusions

In this study, the adsorptivity of the composing materials for MPS, a promising technology for high-throughput drug screening systems, are discussed. As shown in previous studies, the hydrophobic drug cyclosporine A had a greater tendency to be adsorbed on PDMS. Thus, toxicity due to cyclosporine A was decreased in PDMS-MPS but not in COP-MPS. On the other hand, the hydrophilic drugs oxaliplatin and gentamicin had a lower tendency to be adsorbed on PDMS. They did not exhibit any significant differences in cytotoxicity between COP-MPS and PDMS. Therefore, hydrophobic drugs that are easily adsorbed on polymers should be used with COP, which is a low-adsorption polymer. 

COP-MPS has an advantage on PDMS in terms of hydrophobic drug adsorption; however, the adhesive tape interfered with the bonding. We found that the adhesive tape exhibited drug adsorption and cytotoxicity in COP-MPS, although the surface area exposed to the medium was limited. Thus, the material applied for bonding interfaces has an effect on the adsorption and cytotoxicity in MPS, and it is therefore necessary to select the proper method and material.

## Figures and Tables

**Table 1:** Antibodies and dilution ratios for immunostaining.

Antibody	Maker & Catalogue No.	**Dilution Ratio**
ZO-1 Monoclonal Antibody	ThermoFisher 33-9100	1:100
Alexa Fluor^®^ 647 OCT-2 Antibody	Abcam ab205482	1:100
Donkey anti-Mouse IgG (H + L) Highly Cross-Absorbed Secondary Antibody Alexa Fluor^®^ 488	Thermo Fisher A21202	1:400

**Table 2:** Molecular weight and partition coefficient of nephrotoxic drugs [26].

Drug	Molecular Weight	Log P
Cisplatin	300	−2.19
Oxaliplatin	397	−0.47
Gentamicin	1390	−3.1
Cyclosporine A	1200	3.64

## Data Availability

Data sharing is not applicable to this article.
